# Limb occlusion pressure versus standard tourniquet inflation pressure in minor hand surgery: a randomized controlled trial

**DOI:** 10.1186/s13018-023-04000-3

**Published:** 2023-07-28

**Authors:** Thepparat Kanchanathepsak, Natsuda Chua Pukrittayakamee, Patarawan Woratanarat, Tulyapruek Tawonsawatruk, Chanika Angsanuntsukh

**Affiliations:** Department of Orthopaedics, Faculty of Medicine Ramathibodi Hospital, Mahidol University, 270 Rama VI Road, Ratchathewi, Bangkok, 10400 Thailand

**Keywords:** Tourniquet, Limb occlusion pressure, Recommended tourniquet pressure, Minor hand surgery

## Abstract

**Background:**

In minor hand surgery, tourniquet is typically inflated to 250 mmHg. The pressure may be too high and cause unnecessary adverse effects. Limb occlusion pressure plus safety margin or recommended tourniquet pressure (RTP), has been reported as optimal pressure to provide bloodless field in limb surgeries. This study aimed to compare the RTP with the standard tourniquet pressure of 250 mmHg in minor hand surgery.

**Methods:**

A double-blinded randomized control trial was conducted from July to December 2019 and June 2020 to May 2021. Patients were randomly assigned into two groups: RTP and 250 mmHg with 3:1 ratio allocation. The outcomes were measurement of cuff pressure reduction, time to develop of tourniquet pain and discomfort, pain score, discomfort score, motionless and bloodless of operative field determined by the surgeon’s satisfaction.

**Results:**

A total of 112 patients were included, 84 were in RTP and 28 were in 250 mmHg group. Mean of tourniquet pressure was significantly lower in the RTP group (228.3 ± 17.2 mmHg) (*P* < 0.001). Even though, time to develop pain was not significantly different, the RTP group reported significantly less pain and discomfort, according to the pain score (*P* = 0.02) and discomfort score (*P* = 0.017). The RTP group provided better motionless field, while both groups equally created a bloodless field.

**Conclusion:**

The RTP significantly reduced tourniquet related pain and discomfort during minor hand surgeries. It provided better motionless operative field and adequate bloodless field. Therefore, the RTP should be considered as optimal tourniquet pressure for minor hand surgeries.

***Trial registration*:**

TCTR20210519001 (retrospectively registered).

**Level of evidence:**

I.

## Introduction

For limb surgeries, tourniquets are used to create a bloodless surgical field. Although the standard tourniquet pressure is commonly used, it may apply an amount of pressure that is higher than necessary and may cause some adverse effects [1]. The most common adverse effect is tourniquet pain and discomfort, which is reported in up to 60% of patients after surgery [2].

Tourniquet pain is a common complaint for patients in minor hand surgery, many anesthesia techniques, tourniquet position and even if necessity of tourniquet used have been proposed for alleviate this problem.

The Wide-awake Local Anesthesia No Tourniquet (WALANT) technique has become increasingly popular in hand surgery. The benefit of this technique is the ability to assess the active movement during the operation such as flexor tendon repair or fracture fixation [3]. Although, the WALANT has effective local anesthesia technique in hand surgery, however, the approach is difficult for surgeon who has inexperienced including method of injection.

Another useful tourniquet technique is intravenous regional anesthesia or Bier block regional technique which could be performed for most upper extremity surgery, there switching of the tourniquet from the distal cuff to the proximal cuff that eliminates tourniquet pain after 20 min. The advantage of this technique is the procedure time can proceed for up to 90 min, thus there is more suitable for major operation. However, there is a challenging technique and require waiting time for pain subside compared with the minor surgery and some complication was reported of anesthetic toxicity after tourniquet released [4].

Limb occlusion pressure (LOP) is the minimum pressure that can stop blood flow between the proximal and distal parts of the limb, which varies for each person. The LOP plus a safety margin, adding to prevent blood pressure fluctuation, can be used as the optimal tourniquet pressure or recommended tourniquet pressure (RTP) [1, 5, 6].

Currently, a Doppler stethoscope is the gold standard to obtain the LOP [1, 7]. LOP is basically determined by manually increasing the tourniquet pressure until the distal arterial pulse disappears. However, this method is time-consuming and requires operator skill [5, 8]. To overcome these challenges, automatic LOP measurement calculation has been developed. This method measures LOP by automatically adjusting cuff pressure while monitoring distal arterial pulse using a sensor clipped to the finger. The measurement takes approximately 30 s and is user friendly [1, 5].

From previous studies, RTP can sufficiently maintain the quality of bloodless field during operations with lower pressure than standard tourniquet pressure [1, 5, 6]. However, most of them focus on lower extremity surgeries. We hypothesized that the RTP can be applied to minor hand surgeries to provided bloodless surgical field. With limited data on upper extremity, the primary aim of this study was to compare RTP with standard tourniquet pressure in minor hand surgery, in terms of tourniquet pain/ discomfort. Our secondary aims were to evaluate pressure reduction, surgeon satisfaction, quality of bloodless surgical field and complications associated with tourniquet pressure.

## Methods

A double-blinded randomized control trial was conducted at Ramathibodi Hospital from July to December 2019 and June 2020 to May 2021. Patients older than 20 years of age, with an RTP less than 250 mmHg, and who were diagnosed with de Quervain’s disease, trigger finger, carpal tunnel syndrome or carpal ganglion cyst (dorsal) were included. Patients with bleeding diathesis and contraindications for tourniquets (peripheral vascular disease, deep vein thrombosis, severe trauma, infection and peripheral neuropathy) [9] were excluded. All participants gave their informed consent before being included in the study.

The demographic parameters of the patients, including sex, age, body weight, height, arm circumference (mid-point between shoulder and elbow joint), systolic blood pressure, LOP and RTP, were collected. Underlying diseases such as hypertension, diabetes and dyslipidemia were also recorded. The type of minor hand operation was also classified.

Sample size determination was based on mean differences of visual analog scale (VAS) compared between LOP and standard tourniquet. Adding 20 percent of loss to follow up resulting in 116 patients to be included [10]. The patients were randomly divided 3:1 into the RTP group: the control group (standard 250 mmHg). More participants in the RTP group were required for outcomes, safety and detect adverse events [11] and also provide same statistical power for the smaller cost and wound be successfully implemented in practice. While using of the standard treatment was already well-documented. A randomization was conducted by STATA into blocks of four and the assignments were concealed in sealed envelopes. The ethics committee of our institute approved the study.

**Fig. 1:**
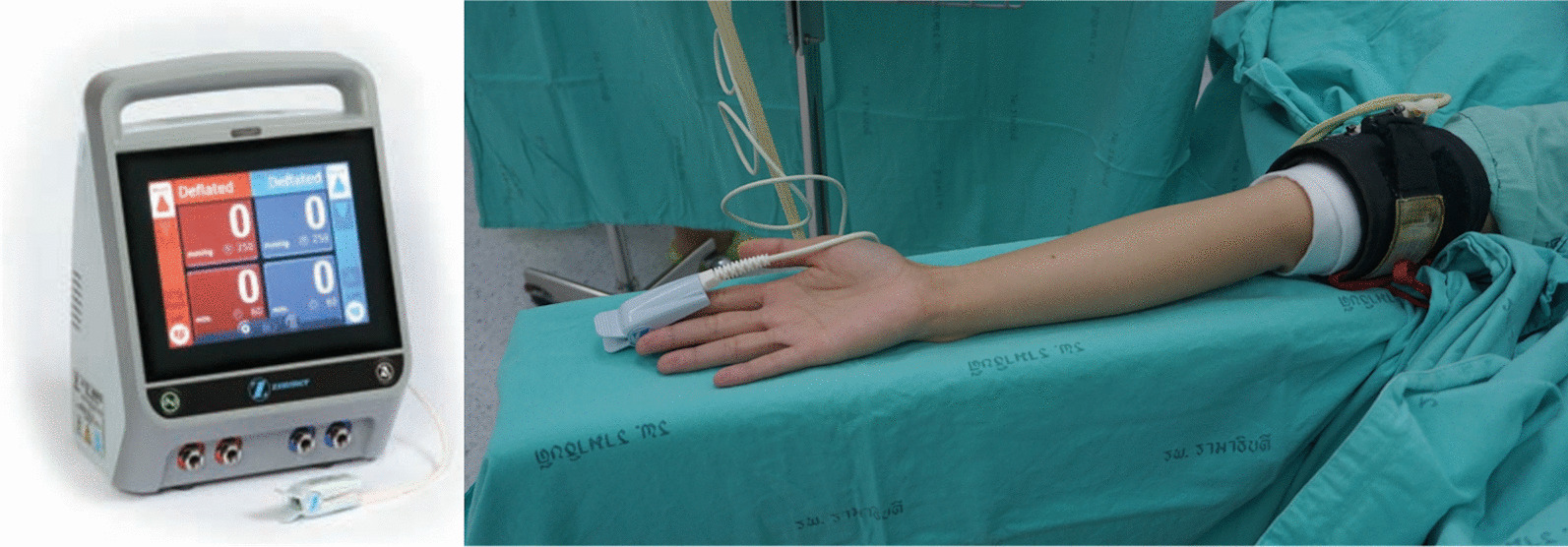
Automatic LOP measurement equipment (ZIMMER A.T.S. 4000)

The LOP was measured for all the patients using automatic LOP measurement equipment (Zimmer A.T.S. 4000, Zimmer Surgical, Inc., Ohio, USA) (Fig. 1). The RTP was defined as LOP plus a safety margin of 50 mmHg for LOP measurements that ranged from 90 to 130 mmHg, 75 mmHg for LOP measurements that ranged from 131 to 190 mmHg and 100 mmHg for LOP over 190 mmHg. LOP plus safety margin more than 250 mm Hg was excluded because it was higher than that of the control group and not generally used in upper limb [6, 12]. This RTP was automatically calculated and displayed by the equipment.

Tourniquet application guidelines were made to control the confounding factors that produced incapable results. The size of the tourniquet was determined by width of the cuff is more than half of arm diameter. Skin under the cuff was protected by 2 layers of 4-inch webril bandage. The cuff was applied on the upper arm which is widest part of the limb to allow as much tissue as possible to lie between the cuff and any nerves or vascular structures susceptible to damage. LOP pulse sensor was attached to the patient’s finger on the limb that the tourniquet has been applied. The corresponding LOP button on the equipment was started for LOP determination. The equipment calculated and displayed the LOP and RTP on the monitor screen approximately within 30 s then pulse sensor was detached from the patient’s finger.

After the operative field was prepared with a sterile technique, 1% lidocaine without adrenaline was infiltrated over the incision site before tourniquet inflation, 2 ml for trigger finger and de Quervain’s release and 5 ml for carpal tunnel release and dorsal carpal ganglion excision. Limb was elevated and 3-inch elastic bandage was exsanguinated from fingers to proximal forearm. Tourniquet pressure was applied to the patient by assigned staff who opened the envelope. RTP was applied to the patients in the RTP group, and 250 mmHg was applied to the patients in the control group. The surgeons, well-trained Orthopaedic resident, was not informed about the amount of pressure applied to the patient.

During the perioperative period, the total tourniquet time was noted. The patient was instructed to inform staff when pain or discomfort (tightness or tense) occurred at the tourniquet site. Time of pain or discomfort began (time to developed pain) were documented. Pain and discomfort were assessed using VAS separately (0–100; 0 = no pain/discomfort, 100 = worst possible pain/discomfort).

Tourniquet was deflated after wound closure and bandage. The surgeon was asked to complete a questionnaire to evaluate the quality and their satisfaction with the surgical field during the operation. The quality of the bloodless and motionless fields was scored as 1 = poor, 2 = fair, 3 = satisfied, and 4 = very satisfied. A poor field was defined as blood or motion occurring and making the operation impossible; a fair field was defined as blood or motion occurring but not significantly disturbing the operation; a satisfactory field was defined as blood or motion occurring with no disturbance to the operation; and a very satisfactory field had no blood or motion occurring. All adverse effects at the tourniquet site after the operation such as coldness, numbness and skin redness were also noted in the questionnaire. One week after the operation, pain, discomfort and adverse events were also monitored.

### Statistical analysis

All statistical analyses were analyses based on intention to treat analysis using STATA version 16.1 (Stata Corp. Texas, USA). Baseline characteristics and outcomes of interest were described based on the mean, median, standard deviation (SD) and percentage. Continuous variables between the two groups were compared, and the differences were analyzed with either unpaired t-test or Fisher’s exact test. For non-normally distributed variables, Mann–Whitney U test was used. *P* value < 0.05 was considered significant.

## Results

**Fig. 2:**
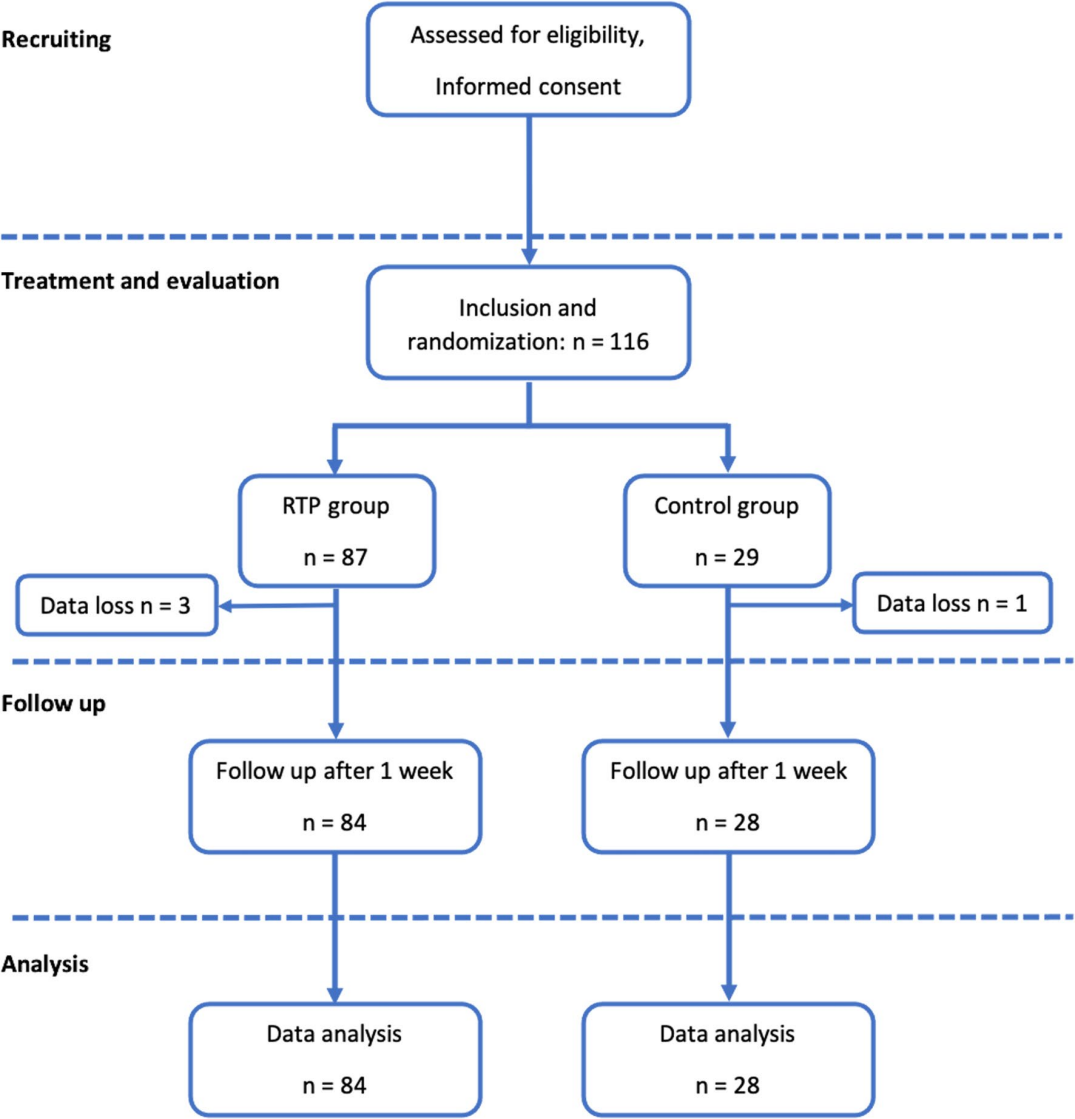
CONSORT flow diagram showed the randomization process. *RTP* recommended tourniquet pressure

One hundred and twelve patients were included in this study: 84 patients in the RTP group and 28 patients in the control group (Fig. 2), two patients in each group had 2 operations in the same time. Considering the baseline characteristics, there were no significant differences between the two groups (Table 1).

**Table 1:** Baseline characteristics

Variables	RTP group (*N* = 84)	Control group (*N* = 28)	*P* value
*Demographic parameters*			
Age (years), mean (SD)	56 (11.7)	55 (9.2)	0.66
Female (%)	64 (77.1)	23 (85.7)	0.43
BMI (kg/m^2^), mean (SD)	24.5 (3.9)	24.7 (4.7)	0.88
Arm circumference (cm), mean (SD)	28 (3.6)	27.6 (3.7)	0.60
Systolic blood pressure (mmHg), mean (SD)	139.6 (18.9)	141.9 (17.3)	0.56
Diastolic blood pressure (mmHg), mean (SD)	74.8 (9.3)	76.8 (10.7)	0.39
Limb occlusion pressure (mmHg), mean (SD)	155.4 (13.7)	156.5 (13.8)	0.71
RTP (mmHg), mean (SD)	228.3 (17.2)	230.9 (16.8)	0.48
*Underlying disease*			
Hypertension (%)	15 (18.1)	8 (28.6)	0.28
Diabetes mellitus (%)	12 (14.5)	5 (17.9)	0.76
Hypercholesterolemia (%)	17 (20.5)	4 (14.3)	0.58
Other underlying disease (%)	17 (20.5)	4 (14.3)	0.58
*Type of operation*			
de Quervain’s disease (%)	10 (12)	2 (7.2)	0.73
Trigger finger (%)	43 (51.2)	19 (67.9)	0.19
Carpal tunnel syndrome (%)	26 (30.9)	9 (32.1)	1.00
Ganglion cyst (%)	2 (2.4)	0	0.30

*RTP* recommended tourniquet pressure, *BMI* body mass index, *SD* standard deviation

For the outcomes of interest shown in Table 2, average tourniquet pressure applied to patients in the RTP group was 228.3 ± 17.2 mmHg compared to standard 250 mmHg in the control group. The difference between these two pressures was significant (*P* < 0.001).

**Table 2:** Outcomes of interests

Outcomes	RTP group (*N* = 84)	Control group (*N* = 28)	*P* value
Tourniquet pressure (mmHg), mean (SD)	228.3 (17.2)	250 (0)	< 0.01*
*During operation*			
Surgeon’s satisfaction score 0–4			
Bloodless field, median (range)	4 (2–4)	4 (3–4)	0.92
Motionless, mean/median (range)	3.9/4 (2–4)	3.8/4 (2–4)	0.049*
Abnormalities, times (%)	3 (3.6)	0	0.57
Tourniquet time (min), median (range)	16 (8–40)	14.5 (8–36)	0.97
Time to pain/discomfort (min), median (range)	8 (1–37)	10.0 (4–29)	0.17
Pain score during operation, mean/median (range)	17.08/0 (0–100)	32.50/15 (0–100)	0.02*
Discomfort during operation, mean/median (range)	17.29/0 (0–100)	33.57/25 (0–100)	0.02*
*After operation*			
Adverse effects after operation			
Nerve damage (%)	4 (4.8)	1 (3.6)	1.0
Tourniquet syndrome (%)	0	0	–
Compartment syndrome (%)	0	0	–
Soft tissue injury (%)	26 (31.3)	10 (35.7)	0.82
*1 week after operation*			
Pain score at 1 week, median (range)	0 (0–0)	0 (0–0)	–
Discomfort at 1 week, median (range)	0 (0–0)	0 (0–0)	–
Adverse effects at 1 week after operation			
Nerve damage (%)	0	0	–
Tourniquet syndrome (%)	0	0	–
Compartment syndrome (%)	0	0	–
Soft tissue injury (%)	0	0	–

*Significant *P* value from either unpaired *t* test or Mann–Whitney *U* test or Fisher’s exact test < 0.05

The time to develop pain and discomfort was 8 min in the RTP group and 10 min in the standard 250 mmHg group. Although pain and discomfort occurred slightly quicker in the RTP group, the difference was not significant. Patients developed pain only 43% in the RTP group, while 61% of controls reported pain (Table 2).

The RTP group reported significantly less pain and discomfort than the standard 250 mmHg group. The mean levels of pain and discomfort (VAS score) in the RTP group were 17.08 and 17.29, respectively, while those in the standard 250 mmHg group were 32.50 and 33.57, respectively (*P* = 0.02 and 0.017, respectively) (Table 2).

Based on the surgeon’s satisfaction evaluation, the motionless field score in the RTP group was higher than that in the standard 250 mmHg group (3.95 and 3.81, respectively) with equal of the median between the two groups which was marginal significant difference (*P* = 0.049). However, there was no significant difference in the bloodless field between the two groups (*P* = 0.92). This study showed no conversion of the patients that had to move from RTP to the standard 250 mmHg group due to bleeding problem during surgery.

Adverse effects at the tourniquet site were reported as coldness, numbness and skin redness immediately after the operation and at the one-week follow-up. However, there was no significant difference between the two groups (*P* = 0.8).

## Discussion

Tourniquet pressure reduction using the LOP technique has been applied in previous studies [1, 5, 6]. However, the scope of these studies was specific to lower limb surgeries. Some studies have attempted to minimize tourniquet pressure in upper limb surgery [10, 13, 14], but studies that reduce pressure using the LOP technique in minor hand surgery are limited.

The present study showed that, in minor hand surgery, using LOP to determine RTP as the tourniquet pressure can significantly decrease cuff pressure from the standard 250 mmHg. Although the difference was only 22 mmHg, however, the lower pressure in the RTP group resulted in significantly less pain and discomfort. The average of RTP in our study was slightly higher than the previous studies that reported an average of arterial occlusion pressure of 200 mmHg because there was not added a safety margin, which is a remain controversial [15, 16]. On the other hand, some study used a safety margin by adjusting the systolic blood pressure of the patients during surgery [17]. However, we recommended to added a safety margin for hemodynamic fluctuation of the patients during operation.

In our study, using the LOP technique to determine RTP, which optimizes cuff pressure, the bloodless surgical field was maintained as same as standard 250 mmHg during the operation, which was comparable to previous studies [1, 5, 6].

Although the average of tourniquet time in RTP group was higher than standard group, 16 min in the RTP group and 14.5 min in the standard group, however, there was no significant difference. An average of the tourniquet time in this study it seems to be slightly higher than usual for minor hand surgery because a surgeon who performed the procedures was an Orthopaedic resident that would be represent a general orthopedic surgeon.

Creating a motionless field was found in the RTP group, which has not been reported in previous studies. This means that the optimal tourniquet pressure provides no difference in creating a bloodless field and provides comparable overall quality of the surgical field. Therefore, the results of our study showed that determining the optimal tourniquet pressure using RTP in the upper limb is practical and satisfactory.

We performed the multiple regression analysis in order to adjust the confounding factors (age, BMI and SBP) and also predict RTP from LOP. The formula was based on the recommended RTP prediction (Predicted RTP = Predicted LOP + Safety Margin). For predicted LOP = 63.41 + (0.31*age) + (0.65*BMI) + (0.42*SBP). Safety Margin was determined by plus 50 mmHg for LOP 90–130 mmHg, plus 75 mmHg for LOP 131–190 mmHg and plus 100 mmHg for LOP above 190 mmHg. This correlation is considered as the optional method to determine RTP when there is no LOP measurement equipment available.

Tourniquet position for upper limb surgery have been debated between using arm and forearm tourniquet. The previous studies showed no significant difference in patient pain, physiological response, tourniquet time, bloodless field or length of operation. However, the arm tourniquet was less obstruction [18] while the forearm tourniquet was subjectively more comfortable [19].

The strengths of this study were that it was a double-blinded randomized controlled trial, with balanced prognostic factors, well-controlled confounders and adequate sample size. However, there are couple limitations including subjectivity of the surgical field evaluation outcome and many surgeons responsible for the operation. Moreover, to measure the LOP the automatic equipment is required.

## Conclusion

In minor hand surgery, using LOP with safety margin to determine RTP as the tourniquet pressure can significantly reduce pain and discomfort, while providing enough pressure to create a high-quality surgical field. With limitation of requiring automatic equipment, the RTP still be a useful option to provide the optimal tourniquet pressure for minor hand surgeries.

## Abbreviations

LOP: Limb occlusion pressure
RTP: Recommended tourniquet pressure
